# Risperidone-Induced Obesity in Children and Adolescents With Autism Spectrum Disorder: Genetic and Clinical Risk Factors

**DOI:** 10.3389/fphar.2020.565074

**Published:** 2020-11-06

**Authors:** Natchaya Vanwong, Nattawat Ngamsamut, Nopphadol Nuntamool, Yaowaluck Hongkaew, Rattanaporn Sukprasong, Apichaya Puangpetch, Penkhae Limsila, Chonlaphat Sukasem

**Affiliations:** ^1^ Division of Pharmacogenomics and Personalized Medicine, Department of Pathology, Faculty of Medicine Ramathibodi Hospital, Mahidol University, Bangkok, Thailand; ^2^ Laboratory for Pharmacogenomics, Somdech Phra Debaratana Medical Center (SDMC), Ramathibodi Hospital, Bangkok, Thailand; ^3^ Department of Clinical Chemistry, Faculty of Allied Health Sciences, Chulalongkorn University, Bangkok, Thailand; ^4^ Yuwaprasart Waithayopathum Child and Adolescent Psychiatric Hospital, Department of Mental Health Services, Ministry of Public Health, Samut Prakan, Thailand; ^5^ Department of Pharmaceutical Care, Faculty of Pharmacy, Payap University, Chiang Mai, Thailand

**Keywords:** obesity, children and adolescents, autism spectrum disorder, risperidone, *ABCB1*, *HTR2C*

## Abstract

**Aims:** Obesity is a significant problem for patients taking atypical antipsychotics. There were two aims of our study. The first aim was to compare the prevalence of overweight and obesity between children and adolescents with autism spectrum disorder (ASD) treated with risperidone with the general pediatric population. The second aim was to investigate the association of the *HTR2C* -*759C*>*T, ABCB1 1236C*>*T, ABCB1 2677G*>*T/A,* and *ABCB1 3435C*>*T* polymorphisms with risperidone-induced overweight and obesity in children and adolescents with ASD.

**Methods:** Body weight and height were measured in 134 subjects. Overweight and obesity in children and adolescents were classified using the International Obesity Task Force (IOTF) criteria. Genotyping was performed by TaqMan real-time polymerase chain reaction (PCR).

**Results:** Our study found that the prevalence of overweight and obesity was significantly higher in children and adolescents with ASD treated with risperidone compared with healthy individuals (*p* = 0.01 and *p* = 0.002). The genetic polymorphisms of *HTR2C –759C>T, ABCB1 1236C>T, ABCB1 2677G>T/A,* and *ABCB1 3435C>T* were not associated with overweight/obesity in children and adolescents with ASD treated with risperidone after adjustment for multiple comparisons by the method of Bonferroni. Additionally, haplotype analysis revealed that there was no significant association between *ABCB1* *3435T-2677T/A-1236T* haplotype and overweight/obesity. In multivariate logistic regression, after adjustment by the Bonferroni correction, there was only the duration of risperidone treatment that was significantly associated with overweight/obesity in children and adolescents with ASD.

**Conclusions:** The findings suggest that children and adolescents with ASD treated with risperidone are at a higher risk of obesity, especially patients with extended treatment with risperidone*.* For the pharmacogenetic factors, *–759C>T* polymorphism of *HTR2C* gene and *1236C>T, 2677G>T/A*, and *3435C>T* polymorphisms of *ABCB1* gene were not likely to be associated with the susceptibility to overweight/obesity in children and adolescents treated with risperidone. Due to the small sample size, further studies with a larger independent group are needed to confirm these findings.

## Introduction

The use of atypical antipsychotics (AAPs) in children and adolescents has increased sharply over the last decade (Comer et al., 2010; Olfson et al., 2010). Risperidone is an AAP approved by the U.S. Food and Drug Administration (FDA) for treating behavioral disturbances in children and adolescents with autism spectrum disorder (ASD) (Hsia et al., 2014; LeClerc and Easley, 2015). A particularly concerning side effect of AAPs is weight gain (Calarge et al., 2009; Correll et al., 2009) as childhood obesity is one of the most significant public health concerns (Chia and Boston, 2006; Güngör, 2014). Weight gain and obesity pose health risks in children and adolescents including sleep-disordered breathing (Bixler et al., 2009), hypertension (Friedemann et al., 2012), dyslipidemia (Friedemann et al., 2012), type 2 diabetes (Goran et al., 2003), cardiovascular disease (Umer et al., 2017), cancer (Weihrauch-Blüher et al., 2019), and reduced lifespan regardless of adult weight status (Must et al., 2012). Obesity also presents a major economic burden (Trasande and Chatterjee, 2009; Wang and Dietz, 2002). Therefore, the prevention and management of overweight and obesity are necessary for children and adolescents undergoing treatment with AAPs.

There is significant inter-individual susceptibility to AAP-induced weight gain in childhood (Correll et al., 2009), yet the pharmacogenomics underlying this relationship is still poorly understood. Several studies have examined the relationship between genetic factors and AAP-induced weight gain. Genetic variations of AAP target receptors have been widely studied for their potential use as genetic markers to predict side effects such as weight gain and obesity. Risperidone is a potent antagonist of the serotonin receptor 5-hydroxytryptamine receptor 2C (HTR2C) (Di Matteo et al., 2002), a proposed target for AAP-induced weight gain (Tecott et al., 1995). Studies in adult patients have shown that genetic polymorphisms in the *HTR2C* gene are associated with inter-individual differences in antipsychotic-induced weight gain. Reynolds and colleagues found that the *HTR2C –759T>C* allele is protective against weight gain in patients with schizophrenia treated with antipsychotics (Reynolds et al., 2002). However, previous studies have failed to establish a consistent effect of the *–759C>T* polymorphism in AAP-induced weight gain (Ellingrod et al., 2005; Park et al., 2008; Tsai et al., 2002).

In addition to target receptors, the efflux transporter P-glycoprotein (P-gp), encoded by the *ABCB1* gene, plays an essential role in antipsychotic drug response. P-gp regulates drug bioavailability by controlling transport across the blood-brain barrier (McCaffrey and Davis, 2012), kidney (Masereeuw and Russel, 2012), and other organs. Risperidone has a strong affinity for P-gp *in vitro* (Boulton et al., 2002), supporting a mechanism by which *ABCB1* gene polymorphisms might influence risperidone pharmacokinetics and weight gain. Interestingly, the *ABCB1* gene is highly polymorphic, with more than 1,200 identified single nucleotide polymorphisms (Fung and Gottesman, 2009). The three most common *ABCB1* polymorphisms include two silent polymorphisms, *c.1236C>T* (exon 12, p.Glu412Glu) and *c.3435C>T* (exon 26, p.Ile1145Ile), and the missense variant *c.2677G>T/A* (exon 21, p.Ala893Ser/Thr), each of which has been shown to significantly minimize P-gp functionality *in vitro* (Salama et al., 2006). Additionally, *2677G>T/A* and *3435C>T* result in decreased intestinal *ABCB1* expression (Hoffmeyer et al., 2000). Moreover, *ABCB1 2677G>T/A* and *3435C>T* polymorphisms were shown to influence risperidone-induced weight gain in patients with schizophrenia (Kuzman et al., 2008).

The aims of this study were 1) to compare the prevalence of overweight and obesity between children and adolescents with ASD treated with risperidone with the general pediatric population and 2) to investigate the association of genetic polymorphisms of the target receptor gene *HTR2C –759C>T* (rs3813929) and efflux transporter gene *ABCB1 1236C>T* (rs1128503), *2677G>T/A* (rs2032582) and *3435C>T* (rs1045642) with overweight/obesity in children and adolescents with ASD treated with risperidone. We hypothesized that children with ASD might be vulnerable to obesity, compared to the general pediatric population. Moreover, considering the role of target receptor HTR2C and efflux transporter P-gp in risperidone pharmacokinetics, we hypothesized that polymorphisms in the *HTR2C* and *ABCB1* genes might impact obesity in children and adolescents treated with risperidone.

## Materials and Methods

### Participants

Between 2012 and 2013, 134 children and adolescents with autism spectrum disorder (ASD) were recruited from Yuwaprasart Waithayopathum Child Psychiatric Hospital, Samut Prakan, Thailand. Patient compliance was confirmed by the nursing staff. All patients 1) were diagnosed with ASD according to the Diagnostic and Statistical Manual of Mental Disorders (DSM-IV) criteria, 2) were medicated with risperidone for more than 3 months, and 3) had a complete record of duration and dose of risperidone treatment. We excluded patients 1) who changed medication or were unable to take medicine regularly, 2) with a known history of serious physical conditions, and 3) who took valproic acid, carbamazepine, and lithium. All patients signed an informed consent document before enrolling in the project. Bodyweight and height were measured cross-sectionally with light clothing in the morning. Age, gender, duration of risperidone treatment, the dose of risperidone, and concomitant medication were recorded. The study was approved by the Ethics Committee of the Faculty of Medicine, Ramathibodi Hospital, Mahidol University (MURA2011/541). After the clinician and researcher described the protocol to the patients, the parents of all children involved in the study gave informed written consent to participate. The authors confirm that all research was performed following relevant guidelines and regulations.

### Blood Collection and Genotyping Analysis

Blood samples were obtained after overnight fasting and were collected by venipuncture into EDTA. Whole blood was used for genotype analysis. DNA was isolated using the MagNA Pure automated extraction system (Roche Applied Science, Penzberg, Germany). Genotyping was performed by allele-specific TaqMan MGB probe 5′ nuclease assay with real-time polymerase chain reaction ViiA 7 system (Applied Biosystems, Life Technologies). TaqMan-based analysis was performed with indicated primers: *HTR2C –759C>T* (rs3813929; assay ID: C__27488117_10), *ABCB1 1236C>T* (rs1128503; assay ID: C___7586662_10), *ABCB1 2677G>T/A* (rs2032582; assay ID: C_11711720C_30)*, ABCB1 3435C>T* (rs1045642; assay ID: C___7586657_20). Each 20 μl PCR mixture contained 4 μl 5 ng/μl genomic DNA, 10 μl TaqMan Genotyping Mastermix, 1 μl allele-specific TaqMan MGB probe and sequence-specific primer kit, and 5 μl DNase-free H_2_O. The thermal cycler program was set up as follows: 95°C for 10 min followed by 50 repeated cycles at 92°C for 15 s and 60°C for 90 s. The allelic discrimination plot was generated by ViiA 7 software (Applied Biosystems, Life Technologies). In this study, we selected single-nucleotide polymorphisms (SNPs) from the HCB (Han Chinese in Beijing, China) database in the International Hap-Map Project (http://hapmap.ncbi.nlm.nih.gov). The SNPs with minor allele frequencies >10% for each gene were selected for this study.

### Classification of Overweight and Obesity

Overweight and obesity were classified using the International Obesity Task Force (IOTF) criteria, which were established using data sets from six different countries (Cole et al., 2000). According to the IOTF guidelines, the international cut-off points for children and adolescents between 2 and 18 years are a body mass index (BMI) of 25 and 30 kg/m^2^ for overweight and obesity, respectively. We compared the prevalence of overweight and obesity in children and adolescents with ASD treated with risperidone with age-matched participants derived from the general child population (Jitnarin et al., 2011).

### Statistical Analyses

Statistical analyses were performed using the SPSS program. Descriptive analysis was used to summarize the clinical characteristics of patients. The Kolmogorov–Smirnov test was used to test for normal distribution with a 95% confidence level (95% CI). Data were conveyed as a median and interquartile range (IQR) due to a non-normal distribution and compared with the non-parametric Mann–Whitney *U* test. Genetic polymorphisms were assessed for concordance with Hardy–Weinberg equilibrium (HWE). Chi-squared analysis or Fisher’s exact test was used to determine the association between categorical measures, including allele and the presence/absence of obesity. Multiple logistic regression analysis was used to evaluate factors significantly associated with risperidone-induced obesity. Analysis of the *ABCB1* haplotype associated with overweight/obesity was performed using the Haploview program v4.2 (Broad Institute, Cambridge, MA, United States). Statistical significance was set at *p*-value < 0.05. Bonferroni’s correction was applied to adjust for multiple comparisons. According to Bonferroni’s procedure, corrected *p*-values (Pc) were calculated by *p*-values multiplied for the following numbers: for genotypes and alleles = 4, for haplotypes = 6, for the multiple regressions model = 9. Then the significance of Pc value was set to be 0.05.

## Results

### Comparison of the Prevalence of Overweight and Obesity Between Children and Adolescents With Autism Spectrum Disorder Treated With Risperidone and the General Pediatric Population Using International Obesity Task Force Criteria

Among the general pediatric population (Jitnarin et al., 2011), 9.2% of children were overweight and 6.5% were obese, whereas 21.6% of children with ASD treated with risperidone from the current study were overweight and 21.6% were obese (Table 1). When classified by age group, no overweight or obesity was observed in children with ASD under the age of 6 years old. Differences in overweight and obesity among children with ASD treated with risperidone compared with the general population were present from 6 years onward. The prevalence of overweight and obesity between children treated with risperidone and the general pediatric population are summarized in Table 1.

**TABLE 1:** Comparison of the prevalence of overweight and obesity in children with ASD treated with risperidone and the general pediatric population using IOTF criteria (*n* = 134).

Overweight					Obesity			
	Healthy children (*n* = 855 from 9,287) (Jitnarin et al., 2011)	Children with ASD (*n* = 29 from 134)	Odds ratio (95%confidence intervals)	*p*-value	Healthy children (*n* = 604 from 9,287) (Jitnarin et al., 2011)	Children with ASD (*n* = 29 from 134)	Odds ratio (95%confidence intervals)	*p*-value
Total	855 (9.20%)	29 (21.60%)	2.85 (1.24–6.56)	0.01*	604 (6.50%)	29 (21.60%)	3.79 (1.53–9.33)	0.002*
Age group								
3–5 years	242 (2.60%)	0 (0.00%)	0.32 (0.03–3.16)	0.37^a^	269 (2.90%)	0 (0.00%)	0.32 (0.03–3.16)	0.3^a^
6–11 years	390 (4.20%)	15 (11.20%)	2.97 (0.91–9.66)	0.06	251 (2.70%)	20 (14.90%)	5.70 (1.59–20.38)	0.01*
12–18 years	223 (2.40%)	14 (10.40%)	5.44 (1.16–25.52)	0.02*	84 (0.90%)	9 (6.70%)	7.45 (0.90–61.73)	0.03*

Data analyzed with the chi-square test.

a Data analyzed with Fisher’s exact test.

* Statistical significance of *p*-value < 0.05.

### Impact of Clinical Characteristics on the Presence of Overweight/Obesity in Children and Adolescents With Autism Spectrum Disorder

A total of 134 children and adolescents with ASD (121 males, 90.3%; 13 females, 9.7%) with a median age of 10.00 years (IQR = 8.58–12.95) were included in this analysis (Table 2). The prevalence of overweight/obesity varied for each age group. The median duration of risperidone treatment was 65.10 months (IQR = 42.10–84.67) and the median daily dose of risperidone was 1.00 mg/day (IQR = 0.50–1.50). Sixty-seven patients (50.0%) received risperidone monotherapy; the remaining 67 (50.0%) received another drug in conjunction with risperidone. We found that age, duration of risperidone treatment, and the dose of risperidone had a statistically significant association with the presence of overweight/obesity. The details of the clinical characteristics and their association with overweight/obesity are described in Table 2.

**TABLE 2:** Impact of clinical characteristics on the presence of overweight/obesity in children and adolescents with ASD treated with risperidone (*n* = 134).

	Outcome			
	Total	Healthy weight (*n* = 76)	Overweight/obesity (*n* = 58)	*p*-value
Gender				
Male, *n* (%)	121 (90.30%)	67 (88.20%)	54 (93.10%)	0.19^a^
Female, *n* (%)	13 (9.70%)	9 (11.80%)	4 (6.90%)	
Age, years (IQR)	10.00 (8.58–12.95)	9.40 (7.55–11.53)	10.95 (9.48–13.90)	0.001^b^ ^,^ ^c^ ^,^ *
Age group				
3–5 years, *n* (%)	7 (5.20%)	7 (9.20%)	0 (0.00%)	0.01^a^
6–11 years, *n* (%)	88 (65.70%)	53 (69.70%)	35 (60.30%)	
12–18 years, *n* (%)	39 (29.10%)	16 (21.10%)	23 (39.70%)	
Duration of risperidone, months	65.10 (42.10–84.67)	55.45 (32.08–79.14)	68.57 (59.92–93.83)	0.01^b^ ^,^ ^c^ ^,^ ^*^
Dose of risperidone, mg/day	1.00 (0.50–1.50)	0.78 (0.50–1.00)	1.00 (0.50–2.00)	0.03^b^ ^,^ ^c^ ^,^ ^*^
Drug regimen				
Risperidone single regimen, *n* (%)	67 (50.00%)	39 (51.30%)	28 (48.30%)	0.69^a^
Concomitant medication, *n* (%)	67 (50.00%)	37 (48.70%)	30 (51.70%)	
Aripiprazole	1 (1.49%)	1 (2.70%)	0 (0.00%)	
Diphenhydramine	1 (1.49%)	0 (0.00%)	1 (3.33%)	
Methylphenidate	52 (77.61%)	30 (81.09%)	22 (73.33%)	
Fluoxetine	6 (8.96%)	2 (5.41%)	4 (13.34%)	
Folic acid	1 (1.49%)	1 (2.70%)	0 (0.00%)	
Atomoxetine	2 (2.99%)	1 (2.70%)	1 (3.33%)	
Sertraline	3 (4.48%)	1 (2.70%)	2 (6.67%)	
Topiramate	1 (1.49%)	1 (2.70%)	0 (0.00%)	

a Data analyzed with the chi-square test.

b Data are shown as median (interquartile range).

c Data analyzed with the Mann–Whitney *U* test.

* Statistical significance of *p*-value < 0.05.

### Association of *HTR2C –759C>T*, *ABCB1 1236CT*, *ABCB1 2677GT/A*, and *ABCB1 3435C>T* Polymorphisms With Risperidone-Induced Overweight/Obesity in Children and Adolescents With Autism Spectrum Disorder

This study investigated the relation of overweight/obesity with *HTR2C –759C>T*, *ABCB1 1236C>T, ABCB1 2677G>T/A*, and *ABCB1 3435C>T* polymorphisms in children and adolescents with ASD treated with risperidone. There was no association of overweight/obesity with *HTR2C –759C>T* polymorphism (Bonferroni corrected *p*-value = 0.48) (Table 3) or with *ABCB1 1236C>T*, *ABCB1 2677G>T/A*, and *ABCB1 3435C>T* polymorphisms (Bonferroni corrected *p*-value = 0.08, 0.08, and 0.36, respectively) (Table 4). Moreover, for gender analysis, *ABCB1* and *HTR2C* genes polymorphisms were not related to overweight/obesity in both males and females (Tables 3, 4).

**TABLE 3:** Association between *HTR2C –759C>T* polymorphism and overweight/obesity in children and adolescents with ASD treated with risperidone (*n* = 134).

	*HTR2C −759C>T*				
	Absence of the T allele (*n* = 108)	Presence of the T allele (*n* = 26)	Odds ratio (95% CI)	*p*-value	Corrected *p*-value^a^
All (*n* = 134)					
Healthy weight (*n* = 76)	64 (84.20%)	12 (15.80%)	1.69 (0.87–3.30)	0.12	0.48
Overweight/obesity (*n* = 58)	44 (75.90%)	14 (24.10%)			
Male (*n* = 121)					
Healthy weight (*n* = 67)	58 (86.60%)	9 (13.40%)	2.25 (0.89–5.71)	0.08	0.32
Overweight/obesity (*n* = 54)	40 (74.10%)	14 (25.90%)			
Female (*n* = 13)					
Healthy weight (*n* = 9)	6 (66.70%)	3 (33.30%)	0.05 (0.28–0.88)	0.23^b^	0.92
Overweight/obesity (*n* = 4)	4 (100.00%)	0 (0.00%)			

Data analyzed with the chi-square test.

a *p*-values after Bonferroni's correction for multiple tests.

b Data analyzed with the Fisher’s exact test.

**TABLE 4:** Association between *ABCB1 1236C>T, 2677G>T/A,* and *3435C>T* polymorphisms and overweight/obesity in children and adolescents with ASD treated with risperidone (*n* = 134).

	*ABCB1 1236C>T*					*ABCB1 2677G>T/A*					*ABCB1 3435C>T*				
	Absence of the T allele (*n* = 23)	Presence of the T allele (*n* = 111)	Odds ratio (95% CI)	*p-*value	Corrected *p*-value^a^	Absence of the T/A allele (*n* = 36)	Presence of the T/A allele (*n* = 98)	Odds ratio (95% CI)	*p*-value	Corrected *p*-value^a^	Absence of the T allele (*n* = 49)	Presence of the T allele (*n* = 85)	Odd ratio (95% CI)	*p*-value	Corrected *p*-value^a^
All (*n* = 134)															
Healthy weight (*n* = 76)	18 (23.70%)	58 (76.30%)	3.29 (1.18–9.17)	0.02*	0.08	26 (34.20%)	50 (65.80%)	2.50 (1.14–5.45)	0.02*	0.08	32 (42.10%)	44 (57.90%)	1.75 (0.91–3.40)	0.09	0.36
Overweight/obesity (*n* = 58)	5 (8.60%)	53 (91.40%)				10 (17.20%)	48 (82.80%)				17 (29.30%)	41 (70.70%)			
Males (*n* = 121)															
Healthy weight (*n* = 67)	15 (22.40%)	52 (77.60%)	2.83 (0.99–8.07)	0.04*	0.16	23 (34.30%)	44 (65.70%)	2.30 (0.98–5.39)	0.052	0.21	27 (40.30%)	40 (59.70%)	1.60 (0.75–3.43)	0.22	0.88
Overweight/obesity (*n* = 54)	5 (9.30%)	49 (90.70%)				10 (18.50%)	44 (81.50%)				16 (29.60%)	38 (70.40%)			
Females (*n* = 13)															
Healthy weight (*n* = 9)	3 (33.30%)	6 (66.70%)	0.80 (0.62–1.03)	0.53^b^	2.12	3 (33.30%)	6 (66.70%)	0.80 (0.62–1.03)	0.53^b^	2.12	5 (55.60%)	4 (44.40%)	3.75 (0.34–41.08)	0.35^b^	1.4
Overweight/obesity (*n* = 4)	0 (0.00%)	4 (100.00%)				0 (0.00%)	4 (100.00%)				1 (25.00%)	3 (75.00%)			

Data analyzed with the chi-square test.

a *p*-values after Bonferroni's correction for multiple tests.

b Data analyzed with the Fisher’s exact test.

* Statistical significance of *p*-value < 0.05.

The most frequently observed *ABCB1* haplotypes for *1236C>T, 2677G>T/A,* and *3435C>T* polymorphisms were identified in patients enrolled in the current study (Table 5). There was no association between the *ABCB1* haplotypes and overweight/obesity in children and adolescents with ASD treated with risperidone (Table 5).

**TABLE 5:** Frequency of *ABCB1* haplotypes in children and adolescents with ASD treated with risperidone with overweight/obesity and with healthy weight (*n* = 134).

Haplotypes C3435T-G2677T/A-C1236T	Haplotype frequencies	Clinical outcome in children with ASD treated with risperidone		*x* ^2^	*p*-value	Corrected *p*-value^a^
		Overweight/obesity	Healthy weight			
TTT	0.350	0.409	0.306	3.042	0.0811	0.4866
CGC	0.261	0.235	0.281	0.700	0.4029	2.4174
CGT	0.193	0.175	0.207	0.418	0.5177	3.1062
CTC	0.075	0.061	0.085	0.521	0.4702	2.8212
CTT	0.072	0.089	0.060	0.823	0.3644	2.1864
TGC	0.042	0.021	0.059	2.380	0.1229	0.7374

Haplotype order: C3435T-G2677T/A-C1236T.

a *p*-values after Bonferroni's correction for multiple tests.

### Multivariate Logistic Regression Analysis of Predictive Factors for Overweight/Obesity in Children and Adolescents With Autism Spectrum Disorder

A multivariate logistic regression analysis was applied to analyze the association of risperidone-induced overweight/obesity in children and adolescents with genetic variables and non-genetic variables. The results demonstrated that the *ABCB1 1236C>T* and duration of risperidone treatment associated with overweight/obesity. However, after Bonferroni correction, only the duration of risperidone treatment was significantly related to overweight/obesity in children and adolescents with ASD (OR = 1.02, 95% CI [1.01, 1.04], Bonferroni corrected *p*-value = 0.009) (Table 6).

**TABLE 6:** Multivariate logistic regression analysis of predictive factors for risperidone-induced overweight/obesity in children and adolescents with ASD treated with risperidone (*n* = 134).

Predictive factors	*β* (SE)	Odd ratio (95%confidence intervals)	*p*-value	Corrected *p*-value^a^
*ABCB1 (1236C>T)*	1.51 (0.59)	4.52 (1.44–14.23)	0.01	0.09
Duration of treatment	0.21 (0.01)	1.02 (1.01–1.04)	0.001*	0.009*

Data are from logistic regression analyses; backward stepwise method. Variables entered on method: HTR2C –759C>T, ABCB1 1236C>T, ABCB1 2677G>T/A, ABCB1 3435C>T, age, gender, duration of treatment, risperidone dose, and co-medication. *β*, regression coefficient; SE, standard error.

a *p*-values after Bonferroni's correction for multiple tests.

* Statistical significance of *p* < 0.05.

## Discussion

The pandemic of obesity and its long-term consequences on healthspan and longevity is a major global challenge. The growing prevalence of obesity in childhood, especially, portends a staggering burden of disease in individuals and healthcare systems in the decades to come (Hruby and Hu, 2015; Skinner et al., 2018). Several studies indicate that risperidone is an AAP that causes weight gain (Calarge et al., 2009; Correll et al., 2009). Moreover, weight gain is a cause of non-adherence with risperidone medication in children and adolescents (Ceylan et al., 2017). Thus, it is critical to mitigating weight gain as a consequence of risperidone treatment.

Our study indicates that the prevalence of overweight and obesity was significantly higher among children and adolescents with ASD treated with risperidone compared with the general pediatric population (9.2% vs. 21.6%, *p* = 0.01 and 6.5% vs. 21.6%, *p* = 0.002, respectively; Table 1), consistent with a previous study (Hill et al., 2015). These results indicate that the different trajectories of weight gain in these patient populations may start in early childhood. Although risk factors for obesity are similar in children with ASD and the general child population (Kipping et al., 2008; Kirk et al., 2010), children with ASD might be susceptible to other risks such as food selectivity (Schreck et al., 2004; Sharp et al., 2013) and a sedentary lifestyle (Bandini et al., 2013; Must et al., 2014). Our results also support a previous study that demonstrated risperidone was related to an average weight gain in children compared with placebo (McCracken et al., 2002). Taken together, the increased prevalence of obesity observed in patients with ASD may be the result of the lifestyle and/or risperidone medication (Calarge et al., 2009; Scahill et al., 2016) or additional undefined factors.

This study assessed the contribution of non-genetic variables on overweight/obesity in patients with ASD, revealing that the age of patients, a higher dose of risperidone, and/or a longer treatment time are related to overweight/obesity in children and adolescents treated with risperidone (Table 2). However, after adjustment for multivariate regression analysis, only the duration of risperidone treatment was found to correlate with overweight/obesity in children and adolescents (OR = 1.02, 95% CI [1.01, 1.04], Bonferroni corrected *p*-value *=* 0.009; Table 6). Our finding is consistent with previous studies in children and adolescents treated with risperidone (Calarge et al., 2012; Martin et al., 2004). Since the pathophysiology of risperidone-associated obesity remains poorly understood, the involvement of non-genetic factors on the risk of obesity in children and adolescents with ASD requires further evaluation.

To the best of our knowledge, this is the first study to examine the pharmacogenetic impact of *HTR2C –759C>T*, *ABCB1 1236C>T, ABCB1 2677G>T/A,* and *ABCB1 3435C>T* polymorphisms on overweight/obesity in Thai children and adolescents with ASD treated with risperidone. The role of the serotonergic system on appetite control has been known for decades (Lee and Clifton, 2010; Feijo et al., 2011). Notably, the HTR2C receptor can regulate satiety and food intake (Lee and Clifton, 2010). Tecott et al. (1995) revealed that HTR2C receptor-deficient mice are overweight as a result of abnormal control of feeding behavior. Moreover, HTR2C receptor antagonists have been found to delay or prevent the onset of satiety, thereby increasing the size of the meal and weight gain (Balt et al., 2011). The risperidone is an antagonist against the HTR2C receptor (Di Matteo et al., 2002), suggesting that *HTR2C* gene polymorphisms might inform risperidone pharmacogenetics. Antipsychotics downregulate mRNA levels of *HTR2C* in rodents (Buckland et al., 1997). In the context of the *HTR2C –759C>T* gene polymorphism, the *–759T* allele is more abundantly expressed than *–759C* (Buckland et al., 2005). Many genetic studies have reported an association of the *HTR2C –759C>T* gene polymorphism with antipsychotic-induced weight gain. Reynolds et al. (2002) found that Chinese patients with schizophrenia with *–759T* allele experienced were significantly protected from weight gain after treatment with antipsychotics for 10 weeks. The protective potential of the *–759T* allele has also been established in a small trial in youths with ASD treated with risperidone for eight weeks (Hoekstra et al., 2010). However, in our study, the *HTR2C –759C>T* polymorphism was not associated with obesity in children and adolescents with ASD treated with risperidone (Tables 3 **,** 6). Several factors could contribute to the discrepancy, such as inconsistencies in study and treatment duration (Hoekstra et al., 2010; Reynolds et al., 2002), ethnicity, study design, and sample size (; Sicard et al., 2010; Gregoor et al., 2011; Lett et al., 2012; Daray et al., 2017). Importantly, subjects in our study were treated with risperidone for at least one year, yielding results consistent with a prior study of children and adolescents with long-term risperidone treatment (Del Castillo et al., 2013).

Risperidone has a strong affinity to P-gp *in vitro* (Boulton et al., 2002) and it is extensively localized in cerebral capillaries forming the blood-brain barrier (Cordon-Cardo et al., 1989). Moreover, an animal study indicated that P-gp in the blood-brain barrier significantly affects the brain concentrations of risperidone and 9-OH-risperidone by limiting their CNS access (Wang et al., 2004). P-gp is encoded by *ABCB1*, a highly polymorphic gene (Ito et al., 2001; Kroetz et al., 2003). *ABCB1 1236C>T*, *2677G>T/A*, and *3435C>T* polymorphisms significantly minimize P-gp functionality *in vitro* (Salama et al., 2006). Since brain penetration of risperidone and 9-OH-risperidone is limited by P-gp (Wang et al., 2004), patients with *1236C>T*, *2677G>T/A*, and *3435C>T* polymorphisms would likely have decreased P-gp functionality in the blood-brain barrier, a lower efflux function for risperidone, and consequent accumulation of risperidone in the brain. Moreover, the variants of these three coding SNPs, at nucleotides 1236, 2677, and 3435 are in high linkage disequilibrium (Fung and Gottesman, 2009; Hoffmeyer et al., 2000). The previous study showed that patients with *ABCB1 1236T-2677T-3435T* haplotype affected the circulating levels of 9-hydroxyrisperidone and the active moiety (Gunes et al., 2008). Therefore, *ABCB1* gene polymorphisms might affect risperidone access to the brain and consequent adverse drug effects such as weight gain. In the present work, analysis of the *1236C>T*, *2677G>T/A*, and *3435C>T* polymorphisms in the *ABCB1* gene have shown that *1236C>T* and *2677T>A* polymorphisms seem to be associated with overweight/obesity in children and adolescents with ASD treated with risperidone. However, after Bonferroni correction for multiple comparisons, none of the variants exceeded a significant threshold (Table 4). Consistently, our haplotype analysis demonstrated that there were no statistically significant correlations for the *ABCB1* haplotypes with overweight/obesity in children and adolescents with ASD treated with risperidone (Table 5). In a multivariate logistic regression model, *ABCB1 1236C>T* SNP seems to be associated with risperidone-induced overweight/obesity in children and adolescents (Table 6). However, the relation of *ABCB1 1236C>T* with overweight/obesity did not survive Bonferroni correction (Table 6). The current study supports prior reports that did not find associations between *ABCB1 1236C>T, 2677T>A*, and *3435C>T* polymorphisms with the risperidone response (Nuntamool et al., 2017), with metabolic abnormality/insulin resistance (Sukasem et al., 2018), or with steady-state plasma concentrations of risperidone or 9-hydroxyrisperidone (Yasui-Furukori et al., 2004). However, here further discrepancies are apparent in the literature, where the *ABCB1 1236C>T* polymorphism showed a significant association with response to risperidone (Xing et al., 2006) and an observed effect of the *ABCB1 2677G>T* and *3435C>T* polymorphisms on risperidone-induced weight gain in patients with schizophrenia (Kuzman et al., 2008). Future studies using larger samples are required to establish unequivocally whether or not *ABCB1* genotypes are related to the development of obesity in children and adolescents treated with risperidone.

Our study has several limitations. First, it was a retrospective, cross-sectional study. Observations in drug-naive patients are required to establish an association of risperidone treatment with weight gain. Second, environmental factors such as food intake and physical activity that affect body weight were not considered in our study design. Third, the size of our sample was relatively small (*n* = 134), since the highest power of sample size that was calculated by *HTR2C –759C>T (rs3813929)* polymorphism was *n* = 590 (Supplementary Table S1). However, our result demonstrated that there was a borderline significant trend of the association between *ABCB1 (1236C>T)* polymorphism and obesity in children and adolescents with ASD (Bonferroni corrected *p*-value = 0.09) (Table 6). The power of sample size that was calculated by *ABCB1 (1236C>T)* polymorphism was *n* = 145 (Supplementary Table S1. Therefore, our results need to be validated with a larger independent sample of patients.

## Conclusion

In summary, our study suggested that children and adolescents with ASD treated with risperidone are at a higher risk of overweight/obese compared to the general pediatric population. The duration of risperidone treatment was a non-genetic factor found to associate with overweight/obesity after adjustment for multivariate regression analysis and Bonferroni correction. For the pharmacogenetic factors, after Bonferroni correction for multiple comparisons, no effect of the *HTR2C –759C>T* and *ABCB1 1236C>T*, *2677G>T/A*, and *3435C>T* polymorphisms on risperidone-induced overweight/obesity was observed. Since our study had a relatively small number of subjects, our results need to be replicated in prospective trials in a larger independent group with the cautious characterization of confounding factors such as food intake and energy expenditure.

## Data Availability Statement

The raw data supporting the conclusions of this manuscript will be made available by the authors, without undue reservation, to any qualified researcher.

## Ethics Statement

The studies involving human participants were reviewed and approved by The Ethics Committee of the Faculty of Medicine, Ramathibodi Hospital, Mahidol University (MURA2011/541). Written informed consent to participate in this study was provided by the participants' legal guardian/next of kin.

## Author Contributions

CS, NV, NaN, and PL designed the research study. PL and NaN diagnosed and recruited the patients with ASD treated with risperidone. NV, NoN, AP, YH, and RS collected the clinical data, performed the experiments, and evaluated the results. NV analyzed the data. NV wrote the manuscript. CS contributed to the discussion and reviewed/edited the manuscript. All authors are accountable for all aspects of the work. All authors approved the final version of the manuscript.

## Funding

This study was supported by grants of the 1) Pharmacogenomics for Autistic Children Project, Khoon Poom Foundation, The Project in Her Royal Highness Princess Ubonratana Rajakanya Siriwatana Bhanawadee, 2) Office of National Research Council of Thailand 3) Faculty of Medicine, Ramathibodi Hospital 4) Mahidol University.

## Conflict of Interest

The authors declare that the research was conducted in the absence of any commercial or financial relationships that could be construed as a potential conflict of interest.
