# Supplementary material for: Risperidone-Induced Obesity in Children and Adolescents With Autism Spectrum Disorder: Genetic and Clinical Risk Factors
Source: Front Pharmacol. 2020 Nov 6;11:565074. doi: 10.3389/fphar.2020.565074 (PMC7677569; doi:10.3389/fphar.2020.565074)
Supplement: Supplementary file 1 [file Table1_v1.DOCX]

**Supplement Table 1.** Sample size calculation of children and adolescents with ASD treated with risperidone.

| Single nucleotide polymorphisms (SNPs) | Minor allele frequency (MAF) | N |
| --- | --- | --- |
| *ABCB1 1236C>T (rs1128503)* | *C* = 0.40 (Nuntamool N, et al., 2017) | 145 |
| *ABCB1 2677G>T/A (rs2032582)* | *T/A* = 0.48 (Nuntamool N, et al., 2017) | 105 |
| *ABCB1 3435C>T (rs1045642)* | *T* = 0.39 (Nuntamool N, et al., 2017) | 151 |
| *HTR2C –759C>T (rs3813929)* | *T* = 0.14 (Luo C, et al., 2019) | 590 |

The sample size needed to assure 99% sensitivity with 95% confidence interval is calculated with the equation; N = [(Z_α/2_)^2^(P (1-P)]/ [(P) (d)]^2^_._

Where: N represents the number of children and adolescents with autism spectrum disorder,

Z_α/2_ represents the value for 95% confidence interval (1.96)

d represents the allowance error of the test (0.20)

P represents the minor allele frequency of *ABCB1* and *HTR2C* genes polymorphisms
 in Thai or Chinese population treated with antipsychotic.
